# Microbial metabolites in nutrition, healthcare and agriculture

**DOI:** 10.1007/s13205-016-0586-4

**Published:** 2017-04-08

**Authors:** Rajendra Singh, Manoj Kumar, Anshumali Mittal, Praveen Kumar Mehta

**Affiliations:** 1 Department of Biochemistry, VP Chest Institute, University of Delhi, Delhi, 110007 India; 2 Mill Hill Laboratory, Division of Structural Biology and Biophysics, The Francis Crick Institute, London, UK; 3 Centre for Molecular Biology, Central University of Jammu, Raya-Suchani, Bagla, Dist. Samba, J&K 181143 India

**Keywords:** Microorganisms, Metabolites, Healthcare, Agriculture, Nutrition, Supplements

## Abstract

Microorganisms are a promising source of an enormous number of natural products, which have made significant contribution to almost each sphere of human, plant and veterinary life. Natural compounds obtained from microorganisms have proved their value in nutrition, agriculture and healthcare. Primary metabolites, such as amino acids, enzymes, vitamins, organic acids and alcohol are used as nutritional supplements as well as in the production of industrial commodities through biotransformation. Whereas, secondary metabolites are organic compounds that are largely obtained by extraction from plants or tissues. They are primarily used in the biopharmaceutical industry due to their capability to reduce infectious diseases in human beings and animals and thus increase the life expectancy. Additionally, microorganisms and their products inevitably play a significant role in sustainable agriculture development.

## Introduction

Microorganisms are of immense importance to environment and essential to all life forms, and are primary source of nutrients and act as chief recycler in environment (Bisen et al. 2012). Microorganisms are present in extremely large sphere of environment and thrive from abyssal zone to stratosphere (at heights up to 60 km) and in a wide range of temperatures ranging from arctic ice to boiling volcanoes (Imshenetsky et al. 1978; Wainwright et al. 2006). These microscopic organisms are used in the preparation of variety of foods and also used as a source of food and feed supplements. For example, amino acids are obtained from *Corynebacterium*, *Brevibacterium* and *Escherichia coli;* vitamins from *Propionibacterium* and *Pseudomonas*; organic acids from *Aspergillus*, *Lactobacillus*, *Rhizopus*, and enzymes from *Aspergillus*, *Bacillus* (Shimizu 2001; Gurung et al. 2013; Mahmood 2015; Sun et al. 2015). Microbes have been recognized considerably for their potential in the development of bioprocess technologies for unhindered production of food products and supplements to meet increasing demand by continuously growing world population. In addition, microorganism-based methodologies do not constitute a major source of pollution, and therefore, are preferred alternative for overcoming serious environmental problems, which arise from the conventional chemical methods.

According to Business Communication Company (BCC), the total global market for microbes and microbial products was estimated at nearly $143.5 billion in 2014, and is expected to reach nearly $306 billion at a compound annual growth rate (CAGR) of approximately 14.6% over the period from 2015 to 2020 (Microbial Products 2015). New technologies for the production of microbial products are replacing synthetic production processes due to technical and economical advantages. These products include nutrition supplements such as, vitamins and amino acids, organic acids, agriculturally important metabolites, enzymes, flavoring agents, coloring agents and pharmaceutical products (Demain 2007). Healthcare was the largest end-user market for microbes and microbial products at about $100.4 billion in 2014, and expected to increase to nearly $111.5 billion by 2015, and over $187.8 billion by 2020. The large size of the healthcare market reflects the importance of microbe-based biopharmaceutical industry (Microbial Products 2015).

Primary metabolites include amino acids, nucleotides, and fermentation end products such as ethanol and organic acids, which are considered essential for proper growth of microorganisms. Microbial synthesis is becoming the dominant and optimal process for amino acid production because of its ease to produce enantiomerically pure amino acids at low cost and ecological acceptability (Sun et al. 2015). Secondary metabolites are organic compounds that form at the end or near the stationary phase of growth, and are not directly associated with growth, development, and reproduction of microorganisms. These products are largely involved in healthcare activities as antimicrobial agents, antiparasitic agents, antitumor, enzyme inhibitors and immunosuppressive etc. (Demain 1999).

The serendipitous discovery of antibiotic penicillin by Fleming in 1929 has drawn the interest of scientists to investigate the therapeutic role of microbial products for combating life-threatening infections. This lead to mass production of antibiotics during World War II by surface culture techniques and the period till 1960 was called as golden age of antibiotics. For the discovery and concept of antibiotics in infectious disease therapy Alexander Fleming, Howard Florey and Emst Boris Chain shared Nobel Prize in physiology/medicine in 1945. Since then, a large number of soil as well as marine microorganisms have been explored for their inexhaustible involvement in pharmaceutical industry. The products derived from microbes are inevitably used to control and cure many infectious diseases acting as antibacterials, cholesterol lowering agents, immunosuppressants, anthelmintics and antiparasitic drugs (Demain and Sánchez 2009).

Secondary metabolites with activities as plant growth stimulants, herbicides and insecticides have also been reported. Some metabolites, such as adriamycin, bleomycin, daunomycin, and mithramycin were used as antitumor compounds (Kieslich 1986). In addition, secondary metabolites are also used as anesthetics, anti-inflammatory agents, anti-coagulants, anabolics, hemolytics, hypocholesterolemics and vasodilatories (Bentley 1997). Different strategies have been considered for effective and overproduction of primary metabolites, where genetic and physiological manipulations have played a significant role. These methods include over-expression of genes involved in metabolite synthesis, knockout of genes involved in degradation, over expression of associated coenzymes and continuous extraction of metabolites from the culture (Tamano 2014).

In this communication, we are precisely illustrating the roles of microbial metabolites in nutrition, healthcare and agriculture with their current industrial status. The role of microorganisms as a potential source of food and feed supplements, and antimicrobial and antitumor properties in healthcare are emphasized.

## Amino acids

The amino acids are building blocks of protein molecules and hence used in dietary and feed supplements of human and animals, respectively (Mahmood 2015). These organic molecules have various role, such as animal feed additives (lysine, methionine, threonine), flavor enhancers (aspartic acid, monosodium glutamate, serine), antioxidants (cysteine, l-tryptophan and l-histidine), as sweeteners (aspartame made from aspartic acid and phenylalanine), and ingredients in cosmetic and medicinal products (Bommarius et al. 1998; Ikeda 2003; Mueller and Huebner 2003; Leuchtenberger et al. 2005; Park and Lee 2008; Ivanov et al. 2014). Additionally, amino acids are suggested as dietary supplements for body building, bruxism, depression, sleep aid, premenstrual dysphoric disorder, attention deficit-hyperactivity disorder, and smoking cessation. Non-essential amino acids can be synthesized by human body but essential amino acids cannot be synthesized in human body but are required for protein synthesis and therefore, dietary supplement is necessary from external sources. Hence, production of essential amino acids at industrial scale using microbial sources is promising and desirable. The worldwide production technology for amino acids is dominated by microbial fermentation and enzymatic processes owing to cost-effectiveness, ecological acceptability and ease to produce enantiomerically pure amino acids (Ikeda 2003; Becker and Wittmann 2012).

The worldwide production of amino acids was about 6.5 million tons in 2014 and is expected to reach 10 million tons of value nearly $35 billion by 2022 (Amino Acids Market 2015). The commercial production through fermentations and enzymatic transformations mainly use *Corynebacterium glutamicum* and *Escherichia coli* to produce l-glutamic acid (monosodium glutamate), l-aspartic acid, l-phenylalanine, l-lysine, l-methionine, l-threonine, and l-tryptophan. l-lysine is a preferred additive to animal feed and approximately, 1.3 million tons of lysine is produced annually through microbial fermentation using *C. glutamicum* (van Ooyen et al. 2012; Sun et al. 2015).

l-Glutamate, extensively used in food and beverage industry as flavor enhancer, was accounted for almost 40% of whole amino acids volume in 2014. The global l-glutamate market was valued over $8.0 billion in 2014 due to increased application in food and beverages industries as flavor enhancer and in pH regulation **(**Amino Acids Market 2015 **)**. l-Glutamate is a vital element of aged or fermented foods, such as cheese and soy sauce. Microorganisms of the genus *Brevibacterium*, *Corynebacterium*, *Micrococcus* and *Microbacterium* are used for the fermentative production of glutamate (Sanchez and Demain 2008; Mahmood 2015). The chief amino acids used as animal feed additives are lysine, methionine, threonine and tryptophan. The increasing demand of these amino acids are coupled with rising meat consumption as these feed ingredients are essential for good health and regulate metabolic processes of the livestock, such as swine, broiler and cattle to gain faster growth and appropriate weight (Amino Acids Market 2015).

Animal feed was reported as the largest consumer of l-lysine and accounted for approximately 92% of total market volume in 2013. The world market of l-lysine is expected to reach 2.8 million tons of value nearly $7 billion by 2020, at a CAGR of 6.0% from 2014 to 2020 (Lysine market analysis 2014). l-glutamate and l-lysine, main food flavoring and feed ingredients, were produced over 5 million tons in 2013 and the industrial market for amino acids is increasing at a yearly growth rate of 6-8% (Becker and Wittmann 2012; Wendisch 2014; Sun et al. 2015). Aromatic amino acids, such as l-tyrosine, l-phenylalanine and l-tryptophan, are vital amino acids for human diet and important precursors for the production of high value by-products (nitric oxide, polyamines, glutathione, taurine, thyroid hormones, serotonin etc.) (Polen et al. 2005; Sun et al. 2015). The leading manufacturers worldwide are Cargill, Novus, Ajinomoto, ADM, Evonik, DSM and Prinova (Amino Acids Market 2015). The microbial technology has made a significant progress for large-scale amino acids production to meet unhindered supply for increasing demands, using constantly improving biotechnology manufacturing methods involving genetic and metabolic engineering and physiologically manipulations (Barker and Campbell 1981; Wang et al. 2001; Hartmann et al. 2003; Znad et al. 2004; Demain 2007).

## Vitamins

Vitamins are essential micronutrients required in trace amount to maintain normal physiological function of the body. These vital nutrients are not synthesized by mammals, and therefore, dietary supplement is necessary from external sources to maintain the balanced metabolism in all living organisms (Shimizu 2001; Gupta and Gupta 2015). Some vitamins are required as coenzymes to facilitate the biochemical reactions catalyzed by the enzymes. Vitamin K is required for normal blood clotting and also to activate receptor to facilitate transcription mechanisms in bone tissues, and to treat osteoporosis (Berg et al. 2002; Bolander 2006). Vitamin A is required as precursor to rhodopsin and other visual pigments, and also associated with specific gene transcription activation that facilitates growth and development (Berg et al. 2002). Vitamins are produced during regular metabolism of microorganisms and widely used as food additives, health supplements, and therapeutic agent etc. Vitamins are produced commercially either through direct fermentation or combined chemical and microbiological processes using appropriate microorganisms (Shimizu 2001; Bhalla et al. 2007). According to a Global Strategic Business Report, the global market of vitamins is expected to reach over $9.0 billion by 2020 due to increasing health awareness and adoption of precautionary healthcare practices (Vitamins 2015).

Riboflavin (vitamin B_2_), a water soluble vitamin, is essential for growth and reproduction in humans and animals. Vitamin B_2_ deficiency results in cheilosis and dermatitis in humans. The annual production was estimated 4.6 million kg and the leading producer of riboflavin are Hoffmann-La Roche (Switzerland), BASF (Germany), ADM (USA), Takeda (Japan) (Demain 2007). The chief microorganisms used in the fermentative production of riboflavin are two closely related ascomycetes, *Eremothecium ashbyii* and *Ashbya gossypii*. *A. gossypii* is an efficient and preferred source of riboflavin production as it can produce 40,000 times more vitamin than required for its own growth. Genetically engineered *Bacillus subtilis* and *Corynebacterium ammoniagenes* are other bacterial species preferred for riboflavin biosynthesis (Survase et al. 2006).

Cyanocobalamin (vitamins B_12_), an anti-pernicious anemia factor, is required in trace amounts (almost 1 µg/day) and produced commercially exclusively by fermentation process using *Propionibacterium shermanii* and *Pseudomonas denitrificans*. These microbes manufacture 100,000 times more vitamin than they require for their own growth (Kusel et al. 1984; Spalla et al. 1989; Demain 2007).

β-Carotene, pro-vitamin A, is required for vision, proper growth and reproduction. The deficiency or malabsorption of vitamin A causes night blindness, changes in the skin and mucosal membranes (http://www.fao.org/docrep/004/y2809e/y2809e0d.htm). The microorganisms *Blakeslea trispora*, *Phycomyces blakesleeanus*, *Mucor circinelloides*, *Rhodotorula* spp. and *Choanephora cucurbitarum* are used for the production of β-carotene. Among these microbial strains, *Blakeslea trispora* and *Phycomyces blakesleeanus* are preferred and used for the industrial production by submerged fermentation due to high yield of β-carotene (Wang et al. 2012; Mata-Gomez et al. 2014).

## Enzymes

Catalytic activities of microbes have been utilized since ancient times for production of bread, wine, and beer. Enzymes derived from microorganisms have drawn significant attention for extensive applications in food, chemical and healthcare industries due to ease of production, stability, and other technical advantages and therefore, microorganisms are utilized in industries as the preferred source of enzymes than other sources of enzymes production, such as animals and plants The use of biocatalytic activities in several industries including food, feed, leather, textiles are increasing rapidly due to time saving process, cost effectiveness, biodegradable nature and environmental friendly characteristics (Gurung et al. 2013; Adrio and Demain 2014; Singh et al. 2016). Besides, microbial enzymes are also involved in the potential degradation of toxic chemical compounds, such as phenolic compounds, nitriles, amines etc. of industrial and domestic wastes (Li et al. 2012; Choi et al. 2015). The biotechnological developments have led to the manipulation of the microorganisms through recombinant DNA technology, protein engineering and their production in appropriate quantities to meet the demand (Liu et al. 2013). According to a market analysis, the global market of industrial enzymes was estimated $4.2 billion in 2014 and is predicted to grow at compound annual growth rate (CAGR) of 7% from 2015 to 2020 (Industrial Enzymes Market 2015). In healthcare industries, enzymes are used in therapeutic management of health disorders caused due to enzyme deficiencies in humans (Vellard 2003; Anbu et al. 2015).

For example, phenylalanine ammonia lyase is used in degradation of phenylalanine in persons with inherited phenylketonuria disorder (Sarkissian et al. 1999). Besides, sacrosidase (β-fructofuranoside fructohydrolase) enzyme is given to facilitate digestion of sucrose in patients with genetic congenital sucrase-isomaltase deficiency as they are incapable of digesting sucrose (Treem et al. 1999). Utilization of microbial enzymes for different purposes in food, pharmaceutical, textile, paper, leather, and other industries are extensive and incessantly increasing over conventional methods owing to higher effectiveness. Table 1 illustrated applications of few enzymes and their function in respective industries (Kamini et al. 1999; Gurung et al. 2013; Adrio and Demain 2014).

**Table 1:** Few microbial enzymes with their source and uses

Enzymes	Source	Industry	Function
Lipase	*Aspergillus niger*, *A. oryzae*, *A. flavus*, *Candida Antarctica*	Dairy Baking Paper Polymer Detergent Textile Cosmetics Leather Healthcare	Faster cheese ripening, flavor Dough stability and conditioning Pitch control Polymerization of lactones, carbonates Fat stain elimination Denim finishing Skin care Degreasing Digestive disorder
Protease	*Aspergillus niger*, *A. flavus*, *B. subtilis*, *Pseudomonas* sp., *Serraita* sp., *Streptomyces* sp.	Cosmetics Leather Textile Paper Beverages	Removal of dead skin Dehairing, bathing, soaking Protein stain removal Biofilm removal Restrict haze formation
Cellulase	*Aspergillus niger*, *Penicillin funiculosum*, *Bacillus* sp., *Trichoderma reesei*	Textile Detergents Paper	Cotton softening, denim finishing Colour clarification Deinking, drainage improvement
Amylase	*Aspergillus* sp., *Bacillus subtilis*, *B. licheniformi*, *B. amyloilquifaciens*, *Streptomyces* sp.,	Beverages Baking Paper Textile Feed Detergents Leather Healthcare	starch hydrolysis Flour adjustment, bread softness Deinking, drainage improvement Desizing Treatment of barley for poultry and calf Starch stain removal fiber splitting Digestive disorder
Laccase	*Bacillus subtilis*, *Pseudomonas aeruginosa*	Textile Cosmetics Polymer Paper Healthcare	Non-chlorine Bleaching, fabric dyeing Hair dye Polymerization of bisphenol A Non-chlorine bleaching, delignification Detoxification

The prospects of enzymes of microbial origin for industrial applications have grown significantly in the 21st century and their valuable contribution in food industry may be used to meet incessantly growing demand of food supply for rapidly growing population. Furthermore, these are used in development of alternative fuel supply to overcome the issues associated with depletion of natural resources and in the development of green environment.

## Organic acids

Organic acids are among the most versatile ingredients in food, beverages, pharmaceuticals, solvents, petrochemicals, textile, detergents, detergents, pharmaceuticals, rubber, perfumes, plastics, dyes and adhesives (Sun et al. 2015). In addition, they are used widely in the production of chemicals that are utilized in the automotive and construction industries. The catalytic potential of microbes is used in commercial production of several organic acids, such as acetic acid, lactic acid, gluconic acid, citric acid (Sauer et al. 2008). Global market of organic acids was estimated at approximately $12 billion in 2014 and is expected to reach over $18 billion by 2023. Increasing demand of vinyl acetate monomer in food packaging industry and high growth of pharmaceutical industry are among major driving factors for huge growth in market (Carboxylic Acid Market 2015).

Citric acid is used for a wide range of applications in food industries, such as acidulant, flavorant, preservatives, sequestrant, emulsifiers, and buffering agent. The global market of citric acid was $2.6 billion in 2014 and is expected to reach $3.6 billion by 2020 at a CAGR of 5.5% from 2015 to 2020 (Citric Acid Market 2015). Incessant growth in the production of citric acid is linked with increasing demands in food, beverages, cosmetic industries and personal care products. About 99% production of total citric acid occurs via microbial processes using surface or submerged cultures and approximately, 70% citric acid of total production is used as an acidifier or antioxidant in food and beverage industry to preserve or enhance the flavors and aromas of fruit juices, ice cream, and marmalades (Lancini 2008). *Aspergillus* sp. and several other yeasts *Candida catenula*, *C. guilliermondii*, *C. tropicalis* and *Yarrowia lipolytica* are employed for the production of citric acid (Kubicek 2001). Among microorganisms, two closely related species of the genus *Aspergillus*, *A. niger* and *A. wentii* are used for industrial production using metabolic engineering (Max et al. 2010).

Itaconic acid is an important building block in the chemical industry for the production of resins, plastics, paints, and synthetic fibers. It is one of the top 12 building block chemicals and a good substitute for acrylic or methacrylic acid, which are used for the production of plastics (Kawamura et al. 1981; Steiger et al. 2013; Otten et al. 2015). The global market of itaconic acid market was estimated $126.4 million in 2014 and is expected to grow at a CAGR of 5.5% between 2015 and 2023. The high demand for itaconic acid is in the manufacturing of superabsorbent polymers (SAP), which are used in diapers, adult incontinence and feminine hygiene products (Itaconic Acid Market 2015). It also finds large application in food packaging, paints and coatings, emulsifiers, pharmaceuticals, detergents, agriculture, herbicides and printing chemicals (Itaconic Acid Market 2015). Several microorganisms such as *Ustilago*, *Candida*, and *Rhodotorula* are capable of producing itaconic acid, but only *Aspergillus terreus* is preferred in commercial production of itaconic acid due to high yield up to 80 g/L (Tabuchi et al. 1981; Willke and Vorlop 2001; Okabe et al. 2009; Steiger et al. 2013).

Lactic acid is utilized extensively in food and beverages, polymer, pharmaceutical, personal care products, and other industries. The poly lactic acid polymer is biodegradable and biocompatible, and used in pharmaceutical industry for the synthesis of prosthetic devices, sutures and internal drug dosing (San-Martin et al. 1992; Chahal and Starr 2006). The poly lactic acid has several applications in packaging, agriculture, automobile, healthcare, electronics, textile, and other industries (Ghaffar et al. 2014; Singhvi et al. 2010; Martinez et al. 2013). The global lactic acid market is expected to reach $3.8 billion by 2020 at a CAGR of 18.6% from 2015 to 2020, whereas the global polylactic acid market is estimated to reach nearly $5.16 billion by 2020, growing at a CAGR of 20.9% for the same period (Lactic Acid Market 2015). L (+) lactic acid is produced commercially through fermentation using lactic acid bacteria (*Lactobacillus* sp.) or fungi (*Rhizopus* oryzae) in submerged culture. Lactic acid producing bacteria are preferred over fungi as the yield of lactic acid is very high through bacterial mediated fermentations. Stereochemically pure L-(+)-lactic acid is solely produced by *Rhizopus oryzae* (Martinez et al. 2013). The global chief manufacturers of lactic acid are BASF SE (Germany), CSM N.V. (The Netherlands), The Dow Chemical Company (U.S.), Teijin Ltd. (Japan), and Nature Works LLC (U.S) (Lactic Acid Market 2015).

The global market for acetic acid is estimated approximately $9.0 billion and expected to reach nearly $12 billion by 2022 at a CAGR of 6.8% from 2015 to 2022 (Acetic Acid Market 2016). Acetic acid is used in the synthesis of other chemical commodities, such as acetic anhydride, vinyl acetate monomer, acetate esters and purified terephthalic acid. These chemicals are further used in wide range of industries such as textiles, construction, automobile, pesticides and food ingredients among others. Vinyl acetate monomer, used in adhesive and sealant industry, was accounted for highest (~33%) consumption of the global acetic acid followed by ester solvents in 2014. A variety of bacteria such as *Acetobacter*, *Gluconacetobacter*, and *Gluconobacter* are used for commercial production (Deppenmeier et al. 2002; Demain 2007). In 2014, the acetic acid production amounted to 12 million tons and is expected to reach a volume of nearly 17 million tons by 2022 at a CAGR of 4.7% during the forecast period (Acetic acid market 2016).

Besides, microorganisms are also used commercially for the fermentative production of gluconic acid by *Aspergillus niger* and *Aureobasidium pullulans*, succinic acid by *Acctinobacillus succinogenes* and pyruvic acid by *Torulopsis glabrata* (Zeikus et al.^1999^; Li et al. 2001; Znad et al. 2004; Anastassiadis et al. 2005; Demain 2007). Major manufacturers in organic acids market are Bioamber, Genometica, GSM cargil and Dow Chemicals.

## Alchohol

Ethanol is a primary metabolite and widely used as biofuel. It is also used as a chemical feedstock for many chemical industries, such as solvent for dyes, oils, waxes, explosives, cosmetics, laboratories and as a disinfectant. According to Renewable Fuels Association (2015), approximately 97.0 billion liters of ethanol was produced worldwide in 2015, and the USA was the largest producer of bioethanol (~56 billion liters), followed by Brazil (~26 billion liters). It is reported that annually over 98% of bioethanol is made from corn. The maximum proportion of total ethanol (~90 to 95%) is produced using microbial fermentation technology (Sarris and Papanikolaou 2016). Recombinant microorganisms, such as *E. coli*, *Klebsiella oxytoca* and *Clostridium thermocellum* are involved in fermentative production of ethanol using different carbon sources (Moniruzzaman and Ingram 1998; Demain et al. 2005). Genetically engineered microbial strain represent >95% ethanol as a fermentation products, whereas wild type *E. coli* produce a mixture of acids (Demain 2007).

Global glycerol market was estimated $1.64 billion in 2014 and is expected to reach nearly $3 billion by 2022 at a growth rate of 7.9%. Global glycerol market size was estimated 2443.9 tons in 2014 and is expected to witness annual gains at over 6.5% from 2015 to 2022 (Glycerol Market Size 2015). Glycerol is used for different applications in foods and beverages, polyether polyols, personal care and pharmaceuticals, alkyd resins, and tobacco humectants. An osmotolerant yeast, *Candida glycerinogenes* has been reported for microbial production of glycerol ~130 g/L with a yield of >60% and productivity of >30 g/L day (Wang and Zhuge 1999; Wang et al. 2001).

## Antibiotics

The antibiotic period began in 1929 with the discovery of penicillin from a fungal sp. *Penicillin notatum* and its commercial production flourished in the 1940s (Demain and Fang 2000). Later, a number of antibiotics have been discovered specifically from fungi and actinomycetes in quest of more effective pharmacological properties and to combat new pathogens. Continuing research in this area led to the discovery of a series of antibiotics, such as cephalosporin, tetracycline, macrolids, ansamacrolids, aminoglycosides, chloramphenicol, glycopeptides, peptide inhibitors, anthracyclins and antifungal antibiotics (Kieslich 1986; Hassan et al. 2012). Nowadays, antibiotics are used for a wide range of applications, such as in chemotherapy, veterinary, plant pathology, food preservation and in research laboratories. These compounds inhibit several pathways, such as nucleic acid synthesis, protein synthesis, cell wall formation, and electron transport pathway (Demain 2007). Some of the microbial species have been reported for their efficient ability to produce a variety of antibiotics. The actinomycetes are responsible for the production of largest proportion (~75%) of antibiotics and the Streptomycetes are the largest producer of antibiotics (Omura 1992; Miyadoh 1993; Zedan 1993; Lazzarini et al. 2000; Berdy 2005). Besides, Streptomycetes are also involved in the potential production of a range of pharmaceutical agents including antiparasitic, anticancer, immunosuppressive and enzyme inhibitors. Antibiotics can be classified in different ways, such as based on their chemical structure, biosynthesis pathway, source, mode of action, route of administration, and their effective range. Tetracyclines, the most extensively used broad-spectrum antibiotics, are used considerably in the treatment of diseases caused by a wide range of microorganism including gram-positive and gram-negative bacteria, protozoan parasites, chlamydiae, rickettsiae and mycoplasma (Chopra and Roberts 2001). Ciprofloxacin, another broad-spectrum antibiotic, is effective in the treatment of urinary tract infections, chancroid, gastrointestinal infection, skin and bone infections and gonorrhea (Davis et al. 1996).

Cephalosporins have a broader spectrum effect against infections and are less likely to be associated with anaphylactic reactions as compared to penicillin (Bhattacharya 2010). Antibiotics market shared around 4.5% of the global pharmaceutical market. The global market of systemic antibiotics was nearly $40.6 billion in 2015 and is expected to reach about $44.7 billion in 2020 at a compound annual growth rate (CAGR) of 2.0% from 2015 to 2020 (Antibiotics 2016).

The global consumption of antibiotic in 2010 was reported highest in India with 13 billion standard units followed by China (10 billion SU) and USA (7 billion SU). The total antibiotic consumption during 2000–2010 was grown by about 35% from 54,000 to 73,500 million standard units. Meanwhile, the consumption of the most common first-line antibiotics penicillin and cephalosporins were increased by 41% and accounted for nearly 60% of total consumption (Van Boeckel et al. 2014; Gelband et al. 2015).

Consumption of two last resort antibiotics carbapenems and polymixins were also increased by approximately 40 and 13% respectively. Carbapenems are used against most difficult Gram-negative infections while polymixins are used to treat multi-drug resistant infections, such as carbapenem-resistant Enterobacteriaceae. Besides, significant increases were also observed for monobactams (>2000%), glycopeptides (>230%), cephalosporins (>90%), and fluoroquinolones (>60%) for the same period. At present, consumption of antibiotics in poultry, swine, and cattle to treat infections and to accelerate animal growth is higher than used for the entire human therapeutic purposes. Worldwide, antibiotics consumptions by livestock was estimated 63,200 tons in 2010 and accounted for nearly 67% of the total antibiotics produced (Pagel and Gautier 2012; Bbosa and Mwebaza 2013; Laxminarayan et al. 2013; Laxminarayan 2014; van Boeckel et al. 2014; Gelband et al. 2015; van Boeckel et al. 2015).

Antibiotics are amongst most frequently prescribed medicine used in healthcare as life saving drugs and hence should be used carefully in treating infections. However, unnecessary and incorrect dosing of antibiotics without proper prescription is the most important factor for antibiotic resistance worldwide. Continuously increasing drug resistance can be prevented through immunization, hand washing, safe food preparation and using antibiotics through proper prescription only when necessary (http://www.cdc.gov/drugresistance/about.html).

Currently, the key manufacturers in the global antibiotics market are Pfizer Inc., Merck and Co., Eli Lilly and Co., Cubist pharmaceuticals, Johnson & Johnson from USA; AstraZeneca and GlaxoSmithKline PLC from UK, and Sanofi-Aventis from France (Antibiotics 2016).

## Antitumor agents

Tumors are generally treated by surgical removal, radiation and chemotherapy. Surgical methods and radiation therapy are inefficient to treat metastatic cancer and therefore, chemotherapy is predominantly helpful in treatment of cancer that has spread to other parts of the body than origin site. According to World Health Organization (WHO), approximately 8.2 million people die annually from cancer, an estimated 13% of all deaths worldwide, and are expected to increase by ~70% new cases of cancer over the next two decades (http://www.who.int/cancer/en/). Many microbial metabolites have been reported for effective anticancer properties in healthcare after the discovery of first anticancer agent, actinomycin by Wakesman and Woodruff. Approximately, 60% of the compounds with anticancer properties are derived from natural sources (Demain and Sánchez 2009). Few microbial products with antineoplastic activities have been illustrated in Table 2. Many of the chemotherapeutic agents used in cancer treatment are secondary metabolites produced by microorganisms, especially of the genus Streptomyces (Demain and Vaishnav 2011; Manivasagan et al. 2014).

**Table 2:** Anticancer agents from microorganisms

Anticancer agent	Source	Type of cancer	Function
Aclacinomycin	*Streptomyces galilaeus*	Lung cancer	Inhibition of nucleic acids and protein synthesis
Calicheamicin	*Micromonospora echinospora*	Acute myelogenous leukemia	Cleavage of DNA
Adriamycin (doxorubicin)	*Streptomyces peucetius*	Uterus and ovarian cancer	Inhibition of DNA replication
Chromomycin A_3_ (toyamycin and aburamycin)	*Streptomyces griseus*	Breast and urinary bladder cancer	Inhibition of RNA synthesis
Actinomycin D	*Streptomyces antibioticus*	Wilm’s tumor in children	Inhibition of RNA synthesis
Taxol (paclitaxel)	*Taxomyces andreanae*, *Nodulisporium sylviforme*	Breast cancer, Kaposi sarcoma	Microtubule depolymerization
Mitomycin C	*Streptomyces caespitosus*	Breast, stomach, oesophagus, bladder	Inhibition of DNA replication
Mithramycin	*Streptomyces plicatus*, *S. argillaceus*, *S. atroolivaceus*	Bone and testicular tumour	Inhibition of of RNA synthesis, mRNA expression and protein synthesis
Daunomycin	*Streptomyces peucetius*	Acute myeloid leukemia, acute lymphocytic leukemia, Neuroblastoma	Inhibition of DNA replication
Bleomycin	*Streptomyces verticillus*	Hodgkin’s disease, squamous cell carcinoma, testicular cancer, pleurodesis	Cleavage of DNA
Neocarzinostatin	*Streptomyces carzinostaticus*	Liver cancer	Proteolysis of histone, degradation of DNA
Thiocoraline	*Micromonospora marina*	Colon cancer	Inhibition of DNA replication
Alteramide	*Alteromonas* sp.	Leukemia, lymphoma, epidermal carcinoma	DNA fragmentation
Epothilone	*Sorangium cellulosum*	Breast cancer	Microtubule depolymerization
Pentostatin (deoxycoformycin)	*Streptomyces antibioticus*	Hairy cell leukemia, acute lymphocytic leukemia, prolymphocytic leukemia	Inhibition of DNA
Streptozotocin	*Streptomyces achromogenes*	Pancreatic islet cell cancer	Inhibition of DNA

The chemotherapy market is currently the fastest growing in the pharmaceutical industry, driven by the magnitude of the disease worldwide and growing understanding of potential therapeutic targets revealed by the molecular genetics assessments of cancer biology.

## Other therapeutic agents

Apart from antibacterial and antitumor agents, other valuable compounds including enzyme inhibitors, immunosuppresants, and alkaloids are of pharmacological importance and also produced by microorganisms.

Immunosuppressant drugs are required to reduce the efficacy of the immune system to prevent rejection of organ transplants and to restrict autoimmune diseases. A number of compounds of microbial origin have been reported for their significant role in suppression of immune response in organ transplant field. Important microbial metabolites, such as cyclosporine A from *Tolypcladium nivenum*, sirolimus from *Streptomyces hygroscopicus*, tacrolimus from *S. tsukubaenis*, mycophenolate mofetil from *Penicillium stoloniferum* and, gliotoxin from *Aspergillus* and *trichoderma* have been reported for their immunosuppressive activities and an effective role in organ transplants (Vezina et al. 1975; Borel et al. 1976; Kino et al. 1987; Demain 2007). In addition, the analogs of rapamycin, such as everolimus (RAD001), tensirolimus (CCI-799) and ARIAD (AP23573) have been developed for their use in heart transplantation and possible role in the treatment of different types of cancer (Demain and Sánchez 2009).

Enzyme inhibitors have received increasing attention for their potential utilization in medicine and agriculture. A number of enzyme inhibitors for different industrial applications have been isolated from microorganisms. Amylase inhibitors (starch blockers) prevent absorption of dietary starches by the body and are useful to control and treatment of carbohydrate dependent diseases, such as diabetes, obesity and hyperlipemia (Jayaraj et al. 2013). In addition, the amylase inhibitors have also been reported for the treatment of rumen acidosis (Banks et al. 2001). Microbial α-amylase inhibitors are paim from *Streptomyces corchorushii* and TAI-A and TAI-B from *Streptomyces calvus* TM-521 (Song et al. 2014). The clavulanic acid is derived from *Streptomyces clavuligerus*. It acts as an inhibitor of β-lactamase and used to overcome antibiotic resistance (Adrio and Demain 2010).

Lipstatin is a pancreatic lipase inhibitor that is produced by *Streptomyces toxytricini* and it obstructs gastrointestinal absorption of fat and therefore plays an important role in the treatment of obesity and diabetes (Rodgers et al. 2012). Acarbose, a pseudotetrasaccharide, contains valienamine which is used to inhibit intestinal α-glucosidase and sucrase. It is produced by *Actinoplanes* sp. SE 50 and useful to reduce starch breakdown in the intestine, and hence used in the treatment of type 2 diabetes mellitus diabetes in humans (Demain and Sánchez 2009). Protease inhibitors, such as antipain, leupeptin and chymostatin have also find important applications in the treatment of AIDS, hepatitis, pancreatitis and cancer. These protease inhibitors are produced by different species of the genus *Streptomyces* (Butler 2008). Enzyme inhibitors of fungal origin are also used against various diseases, such as Alzheimer’s disease, diabetes, cancer and many more (Paterson 2008).

Alkaloids are heterocyclic organic nitrogen containing compounds that have pharmaceutical values including anticancer and anti-malarial effects and are synthesized from amino acids (ornithine, lysine, aspartic acid, phenylalanine, tyrosine and tryptophan) by microorganisms, plants and some animals (Marienhagen and Bott 2013; Wang et al. 2016). For example, nicotine is derived from ornithine whereas ephedrine and morphine are derived from phenylalanine and tyrosine, respectively. The ergot alkaloids were originally derived from ergot, a sclerotium (twisted mat of fungal hyphae). Most of these alkaloids are derived from the *Claviceps sclerotium*. Besides, the alkaloids have also been reported by other fungi including *Aspergillus*, *Penicillium*, and *Rhizopus.* The naturally occurring ergot alkaloids can be divided into lysergic acid derivatives and clavine alkaloids. Ergot alkaloids have been utilized for various diseases, for example ergometrine, ergonovine, and ergotamine for inducing labor in mid-wifery, stopping bleeding after birth, and blocking the sympathetic system for treating migraines, respectively. In addition, the diethylamide derivate of lysergic acid is a powerful hallucinogenic drug for experimental psychotherapy and dihydro derivates of clavinet alkaloid used as strong stimulants of oxytoxic (milk secreting) activity or of uterine contractions. Semisynthetic ergot alkaloids have other major applications as potential therapeutic agents, for example, nigericoline is used in peripheral and cerebral circulation disorder treatment and Lysenyl treats hypertension and migraine. Some other ergot alkaloids have also been reported as pharmaceutical drugs for treatment of prostrate cancer, Parkinson’s disease and lack of milk production after child birth (Schiff 2006; Okafor 2007; Gerhards et al. 2014; Mahmood and Mahmood 2015).

Antiparasitic compounds are used to inhibit the growth and reproduction of parasite organisms that live on the body of humans and animals and can cause serious health problems including death of the host. More than 3200 varieties of parasites have been reported so far from protozoa and helminths. According to WHO, approximately 25% of global population is infected with roundworm (Demain and Sánchez 2009). The significance of antiparasitic drugs can be evaluated by the fact that antiparasitic drug discoverers, Satoshi Omura, William Campbell and Youyou Tu shared the 2015 Novel Prize in Physiology or Medicine for their work on avermectin and artemisinin (World report 2015). Avermectin and its derivatives are extremely effective against river blindness, lymphatic filariasis, and several other parasitic infections while artemisinin is used for most effective treatment for malaria. Omura and coworkers reported eight compounds, which are produced as secondary metabolites by *Streptomyces avermitilis* and were named avermectins (Stapley 1982) and these compounds possessed activity against nemathelminthes and arthropods. Ivermectins is a semisynthetic derivative of avermectin compounds, which is used to control human onchocerciasis and strongyloidiasis (Shikiya et al. 1992). Another derivative selamectin is highly effective against heartworms and fleas in companion animals (Michael et al. 2002). Actinomycetes produce polyether antibiotics, such as monensin, lasalocid A and salinomycin, which are used to control poultry coccidiosis (Shiomi and Omura 2004). The application of anticoccidiales has significantly reduced the mortality and production loss caused by coccidiosis in poultry industry (Westley 1977).

## Biopesticides and plant growth regulators

Chemical pesticides, such as halogenated, carbamate and organophosphorus compounds have been used widely for agriculture system. Their use as pest control results in several problems, such as toxic effect on wild life, human and domestic animals; chemical changes on undesired insects/pests on their predators, parasites and contamination of ground water (Lacey and Siegel 2000; Canan 2013; Nawaz et al. 2016). Biopesticides include biofungicides (*Trichoderma*), bioherbicides (*Phytopthora*) and bioinsecticides (*Bacillus thuringiensis*, *B. sphaericus*) are preferred over conventional pesticides due to biodegradable nature, highly effectiveness, target specificity and less environmental risks (Canan 2013). The global biopesticides market is expected to reach $6.60 billion by 2020 at a CAGR of 18.8% for the period from 2015 to 2020 (Biopesticide market 2015).

Biological pesticides based on pathogenic microorganisms specific to a target pest and pose less threat to the ecosystem. The potential benefits to agriculture and public health programmes through the use of biopesticides are considerable. Over 100 bacteria have been identified as insect pathogens, among them *Bacillus thuringiensis* (Bt), a gram-positive endospore forming bacteria, has got the maximum importance as an insecticidal bacterium to control caterpillar pests, fly and mosquito larvae, and beetles (Argolo-Filho and Loguercio 2014; Nawaz et al. 2016). Bt produces insecticidal endotoxin protein during spore formation that binds to and destroy the cellular lining of the digestive tract, causing the insect to stop feeding and die (Schunemann et al. 2014). The protein kills mainly caterpillars of the Lepidoptera (butterflies and moths), mosquito larvae, and simuliid blackflies. Bt sprays are used on fruit and vegetable crops, on broad-acre crops such as maize, soya bean and cotton (Meadows 1993).

Various subspecies of Bt are used for their different function as bioinsectisides, such as to control beetle larvae (var. *tenebrionis*), caterpillars (var. *kurstaki*, *entomocidus*, *galleriae* and *aizawai*), and mosquito and blackfly larvae (var. *israeliensis)* (Lambert et al. 1992; Milner 1994). *Bacillus sphaericus* is another insecticidal bacterium that has been used to control mosquito species. Certain strains of *Bacillus subtilis*, *B. pumilus*, *Pseudomonas fluorescens*, *P. aureofaciens* and *Streptomyces* spp. prevent plant diseases by outcompeting plant pathogens in the rhizosphere, producing anti-fungal compounds and promoting plant and root growth. They are used against a range of plant pathogens including damping-off and soft rots (Canan 2013; Chatzipavlidis et al. 2013).

Biofungicides have been used in both the phylloplane and rhizosphere to control plant diseases caused by fungi, bacteria or nematodes including some insect pests and weeds. The most common commercial fungal bio-pesticides used in agricluture and forestry are *Trichoderma* spp. and *Beauveria bassiana*. *Trichoderma harzianum* is an antagonist of *Rhizoctionia*, *Pythium*, *Fusarium* and other soil-borne pathogens. *Beauveria bassiana* and *Metarhizium anisopliae* are parasitic fungi found on many insect species. *Beauveria bassiana* has been proved effective in controlling crop pests such aphids, thrips and whitefly pesticide resistant strains. *Metarhizium anisopliae* is used against spittlebugs on sugarcane and grassland and furthermore for the control of locust and grasshopper pests in Africa and Australia. *Coniothyrium minitans* is a mycoparasite applied against *Sclerotinia sclerotiorum* (Chandler et al. 2011; Chatzipavlidis et al. 2013).

Herbicides are widely used in agriculture, industry and urban areas for weed management. With increasing global environmental consciousness, bioherbicides are proved highly effective for weed control in eco-friendly manner. Microbial herbicides can be divided into microbial preparations (microorganisms that control weeds) and microbial derived herbicides. Products based on *Colletotrichum* spp and *Phytophthora* are used commercially against agricultural weeds (Demain and Sánchez 2009; Chandler et al. 2011). The global bioherbicides market was around $973 million in 2015 and is expected to grow at a CAGR of 10.87% for the period from 2015 to 2020. The driving factors for the growth of the market are the ecofriendly nature of bioherbicides, increasing public awareness and lesser development of pest resistance (Global Bioherbicides Market 2016). Research and development in the field of biopesticide applications greatly reduce the environmental pollution caused by the chemical synthetic insecticides and promotes sustainable development of agriculture. Pesticides are employed in modern agriculture to control pests and to increase crop yield.

Plant growth regulators, also called plant hormones are chemical compounds that act as messengers to regulate growth of plants and differentiation of plant cells. There are currently five groups of plant hormones: auxins, gibberellins, cytokinins, abscisic acid and ethylene. These plant hormones work together to regulate the growth and development of cells (Spence and Bais 2015). The plant growth regulators market was estimated to be valued at $1.6 billion in 2015 and is projected to grow at a CAGR of 3.6% from 2015 to 2020 to reach an expected value of $1.91 billion by 2020 (Plant Growth Regulators 2015). Among the types of plant growth regulators, gibberellins accounted for the largest market share followed by cytokinins and auxins. Gibberellins are used on a large scale for extensive production and cultivation of fruits and vegetables and it is mainly involved in early embryo growth, seed germination, stem elongation and flowering. The worldwide consumption of gibberellic acid is approximately 60 tons annually. A number of microorganisms are utilized to produce these hormones (MacMillan 2001; Yurekli et al. 1999; Karadeniz et al. 2006; Tsavkelova et al. 2006). The global leading manufacturers of plant hormones are FMC Corporation and The Dow Chemical Company from USA, Syngenta AG (Switzerland), BASF SE (Germany) and Nufarm Limited (Australia) (Plant Growth Regulators 2015).

## Conclusion

Microorganisms, primary source of nutrients, are essential to all life forms and are efficiently involved in healthcare, agriculture and nutrition. These tiny entities are involved in the preparation of variety of foods and also used as feed supplements. Microorganisms produce antibiotics, antitumor agents, immunosuppressants, alkaloids, and enzyme inhibitors, which are used in the treatment of infectious as well as fatal diseases worldwide and play a key role in reduced mortality rate and better human life expectancy. Application of enzymes has been increasing continuously as microbial enzymes have significant potential for many industries, including pharmaceuticals, food, feed, beverages, detergents, leather processing and paper and pulp to meet demand of rapidly growing population. Pesticides and growth regulators of microbial origin have proved their significant potential in sustainable agriculture development and consequently in the development of green environment.
